# Fiber-Optic System for Monitoring Pressure Changes on Mine Support Elements

**DOI:** 10.3390/s22051735

**Published:** 2022-02-23

**Authors:** Vyacheslav Yugay, Ali Mekhtiyev, Perizat Madi, Yelena Neshina, Aliya Alkina, Farit Gazizov, Olga Afanaseva, Svetlana Ilyashenko

**Affiliations:** 1 Faculty of Power Engineering, Automation and Telecommunications, Karaganda Technical University, Karaganda 100027, Kazakhstan; slawa_v@mail.ru (V.Y.); peri@mail.ru (P.M.); 1_neg@mail.ru (Y.N.); alika_1308@mail.ru (A.A.); 2 Energy Department, S. Seifullin Kazakh Agro Technical University, Nur-Sultan 010011, Kazakhstan; 3 Research School of High-Energy Physics, National Research Tomsk Polytechnic University, 634050 Tomsk, Russia; 4 Department of Economics and Organization of Production, Kazan State Power Engineering University, 420066 Kazan, Russia; fara_gazizov@inbox.ru; 5 Institute of Energy, Peter the Great St. Petersburg Polytechnic University, 195251 St. Petersburg, Russia; eccolga@mail.ru; 6 Basic Department of Trade Policy, Russian University of Economics, 115093 Moscow, Russia; ilyashenkosve@yandex.ru

**Keywords:** fiber-optic system, mining, mine support, accident prevention

## Abstract

The paper presents the developed fiber-optic sensors for monitoring pressure measurement on the elements of mine supports. The sudden destruction of the support leads to the collapse of the mine workings and poses a serious threat to the life and safety of underground workers. A fiber-optic system for monitoring changes in pressures on the elements of mine supports will increase the share of mining automation and reduce the share of manual labor, as well as eliminate measurement errors associated with the human factor. Systematic monitoring of the state of the working elements of the support will allow timely tracking of their deformations caused by an increase in rock pressure on them. Implementation of the system at mining enterprises will expand the use of digital technologies in mining. Timely warning of a mine collapse threat will significantly increase the level of safe mining operations, as well as reduce the cost of supporting mine workings, since elimination of the consequences of destruction is associated with significant material costs. This work presents a developed laboratory testbench that simulates a mine working and elements of an arch support on which are installed the fiber-optic sensors connected to an automated measuring system. The developed hardware and software complex provides the processing of a light spot falling on the surface of a television matrix that is installed at the exit from the optical fiber. The results of visual processing are converted into numerical values, which are used to make a decision about the state of the considered object. In addition to automatic monitoring of the structural integrity condition of the considered object, the system is equipped with a function of a visual display for monitoring results, which makes it possible to track sharp fluctuations and bursts of pressure parameters, based on which the prediction of pre-emergency and emergency situations is performed.

## 1. Introduction

The development of fiber-optic technologies made it possible to create modern information and measurement systems (IMS) that allow tracking a huge number of parameters in real time. This is primarily explained by the fact that the bandwidth of optical fiber (OF) is very impressive, as it has high speed and frequency. Optical fiber is constantly improving, and its cost is decreasing. OF can work in explosive environments, including coal mines, which makes it possible to create the new generation IMS that meet all the requirements of industrial safety. The proposed system is capable of monitoring changes in the rock pressure on the elements of the mine roof support, which will make it possible to prevent its destruction in due time. This will reduce the production costs due to the appropriate reinforcement of mine roof supports in hazardous emergency zones of a mine collapse.

At present, fiber-optic lines have no competitors in terms of data transmission speed and volume [1,2,3]. Through one OF with a diameter of 125 μm and a core of 9 μm, it is possible to transmit all conversations of one large city. Meanwhile, the tensile strength of a thin glass strand of an OF can exceed that of a steel wire of the same diameter. Optical fiber is a rather fragile material. It is based on quartz glass, but it can be easily bent due to its small diameter. When bending the fiber, the minimum allowable radius must be taken into account, which depends on the type of fiber and is set by the manufacturer. If the OF is bent and reaches the value of a critically small radius, cracks are formed in it and it can break. When the OF is bent, a part of the light wave leaves the cladding and is lost [1,2]. Losses of optical power of the light wave (mode) appear, propagating along its core. The principle of data transmission is based on infinite reflection of a mode from the interface between two media with different refractive indices of a light wave [4].

Optical fiber has several advantages. A pulse of light is transmitted through the optical fiber, which is not capable of igniting a hazardous fire environment or causing an explosion in a mine in which the air is gassed with methane [5]. One of the advantages of optical fiber is that it does not generate electromagnetic interference and is not sensitive to it. Many scientific works describe the advantages of optical fiber. Scientists from different countries have been carrying out studies and experiments aimed at developing various sensors [6,7,8,9,10,11,12]. Such fiber-optic sensors (FOS) are used in aviation, space, oil, and gas industries. However, there is a problem of effective control of the geotechnical parameters of mine workings using the existing means that have a mechanical or electrical principle of operation. So far there is no massive use of FOS in mining, and it is impossible to approach this problem one-sidedly. It is necessary to develop new methods for monitoring the parameters of mine workings using fiber-optic sensors to replace mechanical or electrical reference stations (extensometer).

Optical fiber is also used to develop various sensors capable of measuring almost any physical quantity with high accuracy and efficiency [13,14,15]. The use of optical fiber as a pressure and displacement sensor was considered earlier, in [16], and the laboratory studies showed positive results. The idea of using an optical fiber as a sensor is not new. The early developments are described in [17,18], but all possible options for its use have not yet been fully studied. Some properties of OF enable it to be used not only as a sensor but also as a guiding system for data transmission. Accordingly, the efficiency of data transmission increases with minimal power consumption and high noise immunity compared to a radio channel [19,20]. Every year the quality of optical fibers improves and, during data transmission, it allows reducing the loss of a light wave pulse and increasing the length of connection channels. In addition, the semiconductor lasers are currently low noise, and this fact significantly affects the reduction of various kinds of interference [21,22].

Optical fiber is explosion proof and ideal for monitoring rock pressure and displacement in mines and quarries. Point-source fiber-optic sensors are used in quarries to ensure safety and continuous monitoring. Madi et al. [23] presented a sample for studying the entire process of deformation and displacement of a rock mass. The results can be used to optimize work in the quarry and prevent accidents. A physical and mathematical model of a control system for the parameters of an optical signal using an external stimulus sensor based on a diffraction grating with variable characteristics has been developed [24]. Based on the equations, a mathematical model of the diffraction grating was constructed for calculating the resulting intensity and amplitude of interference light passing through the grating. For the lattice model with a round hole, equations were proposed that contained special functions such as the Bessel function, calculated numerically by calculating converging infinite power series and integrals that are not calculated analytically. The degree of accuracy of the developed model is determined by the rigor of the underlying fundamental equations of theoretical and mathematical physics.

As noted earlier in [19,20], the Karaganda coal basin faces an acute problem of rock pressure and rock displacement control. There are no automatic systems capable of controlling the required parameters; periodic inspection of the mine supports by geodetic service workers is required. The specialists of this service perform a visual inspection and control the displacement using reference stations (this is done manually and mechanically) and geodetic instruments. All inspection results are recorded in a journal; reports are stored in paper form. Similar problems exist in coal mines in China and India. In addition to the full automation of mining to reduce the proportion of manual labor in controlling the displacement of rocks and to eliminate the proportion of human faults, the development of digital technologies and the use of FOS are topical issues [25]. Early warning of the impending collapse threat will increase the safety level of mining operations and avoid human casualties [26].

An increase in pressure on the support causes its deformation and damage. Timely notifications of an increase in rock pressure and the occurrence of displacement of rocks make it possible to take appropriate and timely measures to strengthen the support, to prevent its destruction. Destruction of the support not only leads to the formation of blockages but can also cause injury to mine workers. Rock pressure monitoring will reduce the cost of securing mine workings and ensure safe operation [10].

As noted earlier, the operation of optical fiber is completely safe in the mine atmosphere and cannot provoke its explosion [4]. This is confirmed by other sources. Nowadays, the parameters of fiber-optic sensors are superior to those of classical electrical measurement systems. It is recommended to use the fiber-optic sensors with fiber Bragg grating (FBG) in plumbing monitoring and leak detection [27]. In coal mines, the use of this system is not advisable, since it is necessary to control individual points of the mountain range. A sufficiently long, distributed sensor can be easily damaged. A method for detecting the destruction of a rock stratum using warning characteristics was proposed in [28]. The information can serve as a guide for the prevention and control of geological hazards in the working area in high-intensity mining operations; the considered method is simple, convenient, and fast.

A. Ebayyeh et al. [29] created an automatic optical inspection (AOI) system, which performs non-destructive quality control of various products. This method is considered reliable and can replace human inspectors. Rocks are very sensitive to cyclic loads resulting from earthquakes, quarrying, or rock impacts. In [30], parameters were established for determining the fatigue properties and the mechanism of destruction of rocks, which is of decisive importance for the long-term assessment of the stability of building structures in rocks. The mechanics of the continuum of rocks are effectively related with the structural statistics of rock massifs in [31]. The article presents the equivalent continuum models of stress versus deformation, strength, and fracture probability for connected rock mass, which were based on geometric probabilistic models characterizing the structure of the rock mass. The works related to the use of artificial intelligence were analyzed, and possibilities of applying its capabilities in calculations related to the mechanics of rocks were considered [32]. The description of the monitoring system [33] based on the use of radar was considered. The proposed method was very effective for open pits and for predicting the collapse of the sides; however, in the conditions of the workings of coal mines, it cannot be used.

Another work [34] aimed to solve the problem of controlling the geotechnical stability, safety, and integrity of underground infrastructures. This article discussed traditional geotechnical monitoring sensors and described the fiber-optic sensing (FOS) method and recent advances in various FOS-based monitoring systems, including distributed optical Brillouin sensors in the time domain and fiber-optic Bragg grating sensors. The collapse of mine workings in coal mines of China and India was described in [30]. It was stated that without an effective system for monitoring the geotechnical state, the unexpected destruction of mine workings can occur, which leads to injuries and death of mine workers and also entails significant financial losses. The authors stated that roof collapse accounted for 44% of fatalities and 42% of the total deaths in Indian coal mines between 1995 and 2000 [35].

The main advantages of fiber-optic sensing (FOS) are considered in [36,37]. It is an excellent alternative to electrical sounding, a completely intrinsically safe system for monitoring rock pressure and rock displacements. There is experience in using two-level cable extensometers at coal mines in Great Britain and Canada. This is analogous to reference stations (signaling devices), which are installed in the roof of the mine every 20–30 m [38]. Extensometers with a sound sensor are installed immediately after roof anchoring in mines in South Africa that are not hazardous due to the sudden release of dust and gas [39]. However, the use of electrical devices in coal mines is very limited, since an electric spark can cause an explosion. There is information about laboratory tests of anchors with strain gauges in terms of distributed axial and bending deformation along the length of the anchor bolts; but the use of electric current limits the application of such anchors in coal mines [40]. Another problem is associated with the fact that electrical strain gauges are very sensitive to water impact and must be properly protected from a humid environment [41]. To understand the roof erosion process in coal mines in China, a field experiment was conducted using an integrated, real-time monitoring system with extensometers and sensors to measure the deformation of the rock mass caused by mining [42].

The fiber-optically instrumented rock strain and temperature strips (FROSTS) were developed and tested at the mine [43]. The high-strength optical fiber was coated with stainless steel, which has high deformation transfer characteristics, which allows it to be connected with a concrete anchor with uniform deformation. This demonstrated the possibility of using fiber for theoretical and experimental monitoring of the formation motion [44]. The results of safety studies using the conditions of stability of the mountain range are presented in [45]. The results of an innovative analysis method based on a combination of distributed fiber-optic sensors, digital photogrammetry using unmanned aerial vehicles, and topographic and geotechnical monitoring systems are shown. O. Kamenev et al. proposed a method for determining the direction of deformation of a fiber-optic strainmeter [46]. The method is based on the use of a two-channel scheme of a fiber-optic Mach–Zehnder interferometer. For example, the non-parallelism of the loading plates can lead to a significant variation in test results, which will be perceived as the variability of rocks [47]. The paper proposed changing the current testing standards towards better control of loading machine performance and equipment accuracy. It is possible to identify pre-existing micro-cracks in the rock that were not detected by analysis of thin sections or ultrasonic measurements using computer tomography (CT) since they can affect the test results, which is important for obtaining reliable data. G. Karthik et al. provided a detailed overview of available low-cost wireless slope monitoring systems for quarries [48]. G. Chen et al. reported the use of a global positioning system (GPS) for monitoring a coal mine, which uses a combination of open pit and underground mining [49]. The GPS monitoring system has proven to be effective in reducing geological hazards in mining areas.

By analyzing the experience of creating FOS, we can conclude that OF can be used simultaneously as a sensing element and as a guide for a data transmission system. OF is completely safe for use in coal mines. Based on OF, it is possible to create a distributed and quasi-distributed monitoring system for rock pressure and rock displacement. Therefore, it is relevant to conduct scientific research for the development of a prototype of a fiber-optic system for monitoring pressure changes on the shaft support elements.

**Figure 1:**
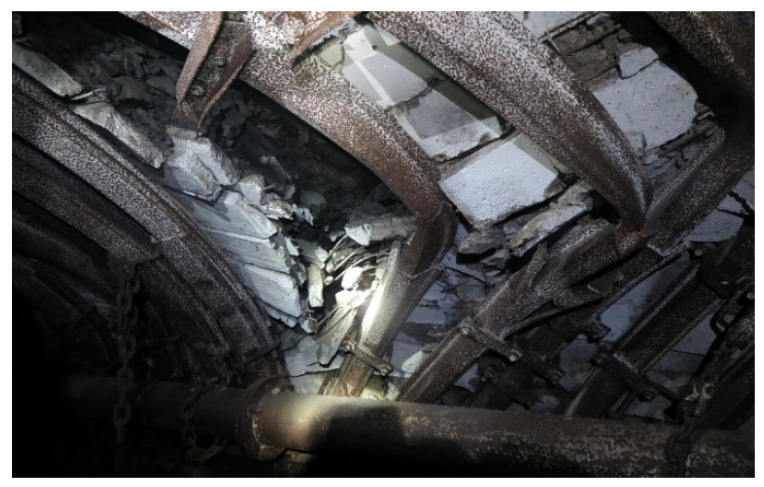
The destruction of the mine support under the change of rock pressure.

Figure 1 shows the destruction of the mine support and the formation of the threat of rock fallout. The photographs were taken at the coal mine of ArcelorMittal Temirtau JSC (Karaganda, Kazakhstan). The photographs were recorded using an explosion-proof camera, since it is prohibited to use common cameras. The photographs were taken by the engineers of the mine surveyor service. Achieving better quality was not possible due to the explosive conditions of the coal mine and the lack of sufficient lighting. The arrows show the results of the destruction of the support.

**Figure 2:**
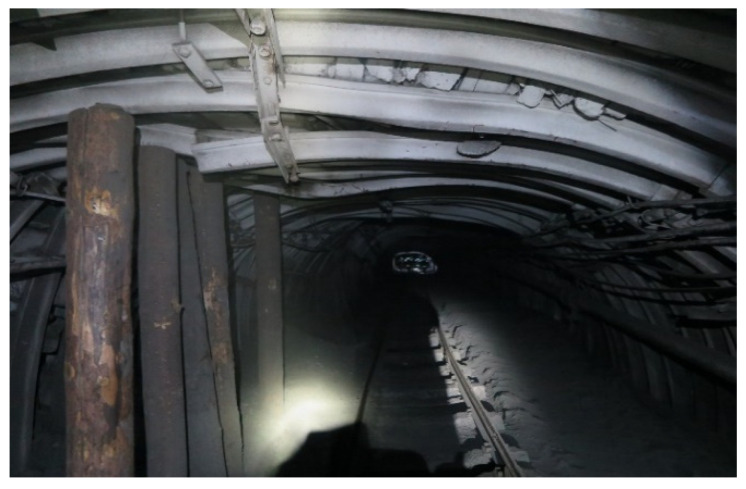
Temporary supports from wooden posts.

Figure 2 shows the picture when temporary supports from wooden posts were installed to prevent further destruction of the support. This situation is dangerous for mine workers and has a threat of collapse of the vault and blockage of the exit from the mine. This picture illustrates the elimination of the consequences of an increase in rock pressure and displacement of rocks. With timely notification of a change in the geotechnical situation of the roof layers of a mine working, these consequences could be avoided.

The arch support can be strengthened in a timely manner by additional elements or supports, and these measures can avoid its destruction. Therefore, additional material costs can be avoided.

Figure 1 and Figure 2 show the KMP-A3 support made from the SVP-22 profile, which is called a flexible, arched, three link with interchangeable profiles. The structural flexibility of this support is up to 240 mm. Depending upon the section of the working, the profiles SVP-17, SVP-22, and SVP-33 were used. This support is widely used in the coal mines of ArcelorMittal Temirtau JSC. The support is made of steel St.5ps; the bearing capacity is up to 330 kN per frame. The photo shows that the profile could not withstand the rock pressure and broke; the place of the rupture is shown by an arrow.

**Figure 3:**
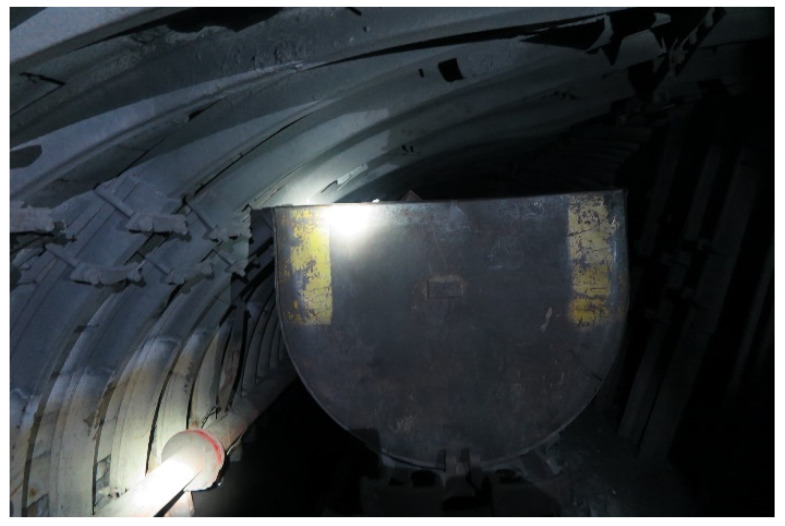
The problem of trolley passage with changed support profile.

Figure 3 shows how the metal arch support is compressed when the rock pressure changes. Since the mine has very limited space, the slightest change in its cross section makes it difficult for technological transport to pass.

This study aimed to develop a laboratory sample of a system for measuring the pressure of rock formations based on fiber-optic sensors. In the future, a working industrial device for scaling and use at the coal mines of ArcelorMittal Temirtau JSC will be created. For the implementation of this system at the coal mines of ArcelorMittal Temirtau JSC, it is necessary that the fiber-optic automated measuring system can control the pressure on the shaft support elements in an explosive environment, has a simple design, and does not significantly increase the cost of one measurement point.

Currently, the mines of ArcelorMittal Temirtau JSC do not have automatic systems for monitoring the geotechnical state of mine workings, which perform the real-time control and meet the safety requirements of coal mines. There are also no promising rock pressure and displacement control systems that could be considered for implementation. The introduction of a fiber-optic automated measuring system (FAMS) will avoid not only additional financial costs but also human casualties. The existing system for monitoring rock pressure and displacement of seams, based on periodic readings from mechanical reference stations with the participation of a person, is not effective. The literature analysis showed that automatic systems for monitoring rock pressure and displacement of seams have not yet been introduced at the coal mines in Russia, Kazakhstan, China, India, and other countries. In this case, control is carried out using reference stations, which are analogous to extensometers.

## 2. Materials and Methods

Taking into account all the features of the structure of the mine workings and arch support, a laboratory testbench was specially designed. When creating the testbench, a model method was used. A material model was created with the maximum possible preservation of the physical nature of the original; its attachment design was close to the original design of the arch support. Metal profiles that imitate the arch support were used. The study was carried out by measuring additional losses during mechanical action on OF and bending formation. The method was based on a change in the properties of light when passing through an optical fiber under mechanical action on it and the effect of light transmission. In the case of a microbend, the effect of photoelasticity arises [19], which leads to a change in the refractive index between the cladding and the core. In the case of a microbend, additional losses occurring in the optical fiber under mechanical action on it increase and changes in the intensity of the light wave and the phase of its propagation also occur. The phase change can be converted to amplitude changes. Accordingly, upon mechanical action on an OF, the properties of the light wave are changed, passing through the OF core and incident on the surface of the photodetector, which records all changes. Further, the data processing unit records the numerical value. In this stand, instead of a well-known photodetector, a high-resolution television matrix CMOS was installed at the output of the optical fiber with a graphic processor of one measurement channel for pre-processing of high-resolution signals. At the end of the optical fiber, a fiber-optic connector of the SC type with a ferrule with a diameter of 2.5 mm was installed, with the Ultra Physical Contact (UPC) connector polishing. This made it possible to form a clearer light spot, unlike the case when the end was cut with a knife for OF cutting. During the setting process, the focal length between the end of the connector and the surface of the photomatrix was determined, as shown in Figures 8 and 9. This setting allowed the hardware–software complex to record all changes in the light spot when OF was under exposure.

Combining different methods will create a fiber-optic sensor (FOS) that operates based on a completely different principle. FAMS was built according to the well-known quasi-distributed type, since it was necessary to control individual points of the mountain range. The use of a fully distributed system has several disadvantages associated with the risk of damage to the sensor and the failure of the entire system. The FOS was based on the known design of a mechanical reference station. This design was simple and had low production cost, but it cannot transmit data over a distance. A mechanical reference station requires periodic visual inspection to read the parameters. The mechanical reference station has been used in coal mines for several decades and has proven itself well, with a cost of about $100. Over the past 50 years, it has not been possible to develop and implement a fully automated system for monitoring rock pressure, since strict requirements for explosion safety are imposed on such devices. Since the sensors have to operate in an explosive environment, it is not possible to use electrical signals and voltages to power them. This can create the danger of an explosion in the atmosphere since methane gas may be present. In this case, the FOS will have a mechanical reference station in a similar design, which will significantly reduce its cost. The complication of the design will lead to the price increase (exceeding $100). FOS cost is one of the advantages for its further implementation. There are several technical solutions that were used in the development of FOS, aimed at reducing the cost of FAMS in general. The proposed FAMS does not use optical interferometry or reflectometry, fiber Bragg gratings, or long-period fiber gratings. The abandoning of the well-known method of optical interferometry was due to the dependence of the phase of propagation of the light wave on temperature; so, a temperature change by 1 °C could cause a false alarm in the absence of mechanical action on the sensor. The use of a distributed FAMS based on optical reflectometry or Bragg gratings is specified by higher cost of one measurement point, as well as the peculiarity of the distributed system operation associated with the use of one OF. Considering that it can be damaged during the operation of technological equipment and its motion, the production will remain without a pressure control system. One should also take into account the specifics of the placement of sensors in the hole drilled in the roof of the mine; this is a quasi-distributed measurement system. The proposed quasi-distributed FAMS has a new principle for constructing and processing data. A quasi-distributed FAMS can have an unlimited number of channels. The length of one measuring channel can be up to 20 km, which is quite enough for any coal mine. As the sensitive sensor of the quasi-distributed FAMS, we used a quartz, single-mode OF of the G.652 standard (Corning, USA) with a diameter of 9/125 μm. The profile of the light spot at the OF output is a stepwise one, which can be described by the Gauss distribution law. These well-studied properties are used in telecommunication systems. All the necessary information can be found in previously published materials that describe the properties of an OF and the process of a light wave propagation in OF [50,51].

At the OF output, a high-resolution television matrix was installed, on the surface of which a light spot fell. The spot contained a lot of noise and was not informative, which is typical for single-mode fiber. This caused several difficulties in video processing and analysis. This property of the OF was used to construct the quasi-distributed FAMS, since this method of analyzing a light spot is not suitable for a distributed system. The resulting image was computer analyzed. The number of formed pixels of white and black colors on the surface of a television matrix and their transition from one state to another were analyzed. Further, the microprocessor performed calculations and output the number of pixels in real time. The number of white pixels increased as the pressure on the OF increased. This fiber is used to make fiber-optic cables and is mass produced, which provides a low cost, in the range of USD 10 per km. OF was simultaneously used as a sensor and a data transmission line, which eliminated the use of electrical signals in the measuring section. Since all work with electric devices generating an electric arc is prohibited, the connection of the FOS was carried out using standard optical connectors and adapters of the SC or FC type. Accordingly, welding work in an explosive atmosphere of the mine will be completely excluded. Unlike a welded joint, optical connectors create large losses at the connections, which can reach 0.2 dB at 0.03 dB for welded joints. This was not critical, since the distance from the data processing unit, which was installed on the surface, to the FOS was from 3 to 10 km, and the incoming light wave was powerful enough to be perceived by the TV matrix.

**Figure 4:**
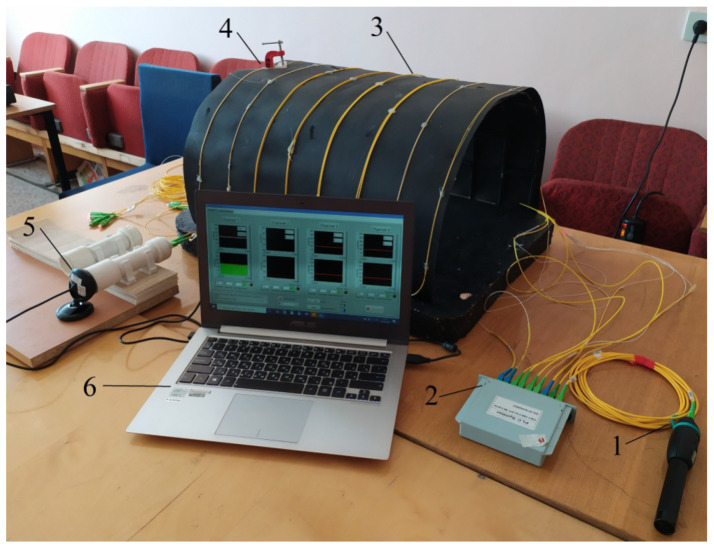
A laboratory prototype of the fiber-optic system of monitoring pressure changes on the mine support elements: (**1**) radiation source, (**2**) optical splitter, (**3**) optical fiber, (**4**) clamp and place of microbend, (**5**) TV matrix with a graphic processor of one measuring channel for preliminary processing of signals, and (**6**) personal computer.

To test the fiber-optic system of monitoring pressure changes on the elements of the mine support, a laboratory testbench was developed. It is shown in Figure 4. The positions are indicated in the following order: (1) radiation source, (2) optical splitter, (3) optical fiber, (4) clamp and place of the microbend, (5) TV matrix with a graphic processor of one measuring channel for preliminary processing of signals; and (6) personal computer.

The photo shows an imitation of a mine working with arched support elements. The optical fiber was glued on top of thick cardboard that simulated the boundaries of the working and the rocks themselves. Pressure on the OF was simulated using a screw clamp. A G652 single-mode OF was used as a sensing element. The software was installed on the laptop, and all the measurement results are stored on the hard disk. The program had several windows for setting up its operation and visualizing the measurement results.

The proposed method for monitoring rock pressure and displacement of roof rocks was based on the control of the numerical value of additional losses, which were represented by a change in the intensity of a light wave incident on the surface of a high-resolution television matrix. The hardware-software complex performed intelligent processing of the light spot image and controlled the changes of the intensity of individual pixels. The computer screen clearly showed how the light spot changed, especially at the interface between the core and the cladding of the optical fiber. Changes occurred when the OF was affected. The incident light on the surface of the television matrix contained a significant amount of noise. This was a problem that had to be dealt with using software algorithms for recognizing the increase and decrease of noise.

As mentioned earlier, noise negatively affected the measurement process, but the software monitored the dynamics of the change in the shape of the light spot and could separate fluctuations caused by external factors (for example, thermal impact on the optical fiber) from useful signals. Since the developed FAMS did not use the well-known methods of the optical interferometer and the Bragg grating, no negative effect on the measurement process was identified. During the experiments, the temperature in the laboratory varied from 20 to 26 °C, which met the requirements of the Rules for Safety and Labor Protection in the Mining Industry of the Republic of Kazakhstan. In the course of laboratory experiments, no negative effect of thermal noise on the operation of the FAMS was revealed. The thermal noise level was negligible and did not affect the measured data. Larger fluctuations in temperature can cause negative impact since the refractive index of the OF changes. The hardware–software complex could distinguish the thermal effect on the OF from the mechanical effect since these were parameters that were different in terms of the rate of change.

The system operation was partly based on the effect of photoelasticity and the change in additional losses upon mechanical action on the OF. An important point in the fight against false alarms is the ability of the system to stepwise change its sensitivity. Initially, the system was set to maximum sensitivity to monitor the initial displacements and alert the operator, after which the parameters were automatically coarsened to accurately capture the displacement and eliminate false measurements. The use of intelligent processing of the results of changing the image of the light spot, namely, the change in the intensity of individual pixels and the rate of change in the derivative of the intensity of the light wave in time, made it possible to increase the measurement accuracy and exclude false data from the measurement process. Numerical parameters were visualized on the computer screen. The program could track sharp fluctuations and surges in pressure parameters while an alarm was triggered.

The program allowed monitoring the change in pressure in four channels at the same time. The InGaAs semiconductor laser (Laser launcher level CLASS IIIB) with a wavelength of 650 nm ± 10 nm was used. The laser power was 20 mW. An optical splitter was used to divide the light wave into four beams of equal power. A ray of light passing through the optical fiber fell on the surface of the television matrix. The integrated graphics processor preprocessed the data and transferred it to the computer.

**Figure 5:**
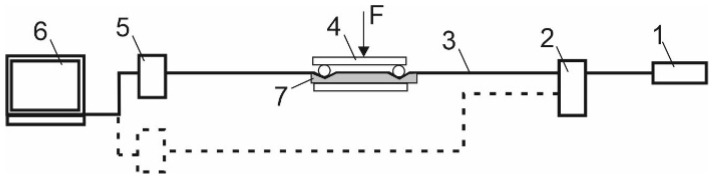
A scheme of fiber-optic measurements: (**1**) radiation source, (**2**) optical splitter, (**3**) optical fiber, (**4**) clamp and a place of microbend, (**5**) CMOS photomatrix with a graphic processor of one measurement channel for signal preprocessing, (**6**) personal computer, (**7**) soft padding.

Then, the software completed the processing of the pressure measurement data. Numerical parameters of measurements were visualized on the computer screen. The program monitored sharp fluctuations and surges in pressure parameters, triggering an alarm. In the case of sharp fluctuations in pressure, the operator received a sound signal and the indication turned on. The measurement diagram is shown in Figure 5, where 1 is a radiation source, 2 is an optical splitter, 3 is an optical fiber, 4 is a clamp and a place of a microbend, 5 is a CMOS photomatrix with a graphic processor of one measurement channel for signal preprocessing, 6 is a personal computer, and 7 is a soft padding.

When developing the FAMS, it was taken into account that constant changes in the propagation phase of a light wave cause interference. An important point is the stability of the coherence parameters of the radiation source; so, it must have high stability with a low level of ripple. The laser radiation power is changed by changing the supply voltage, which is necessary when adjusting the FAMS at different distances to the measurement point. Degradation of the laser and its failure are possible when the supply voltage drops or exceeds its nominal value.

If the connection with a particular FOS is disrupted when the fiber-optic cable is damaged, a warning alarm will be triggered. Under mechanical action on the optical fiber, a microbend occurs. This leads to changing the properties of the light wave passing through its core, especially the parameters of the intensity and propagation phase. Bending also increases the numerical values of additional losses, which are fixed by the program. Accordingly, the more the clamp was tightened, the more the additional losses in the optical fiber increased and the higher the pressure readings rose. A strain gauge was placed under the OF, which was connected to an analog-to-digital converter, which made it possible to measure the applied pressure force. The compression force of the developed clamp was pre-calibrated from 0 to 30 N and the required number of turns of its screw was set to achieve the required load. The additional loss of the light wave passing through the core of the OF increased proportionally to the increasing pressure on the OF. The photomatrix recorded all the changes in the shape of the spot and transmitted the information to the personal computer.

In the future, it is planned to use this system at the coal mines of the Karaganda coal basin. They are hazardous for sudden outbursts and explosions of coal dust and methane gas. The experience of other countries, which have mines of similar danger (in particular, China), was personalized. Based on this, the optical fiber was used as a sensitive element and a guiding system for transmitting measurement data, which excluded the use of electrical signals [18].

## 3. Results

**Figure 6:**
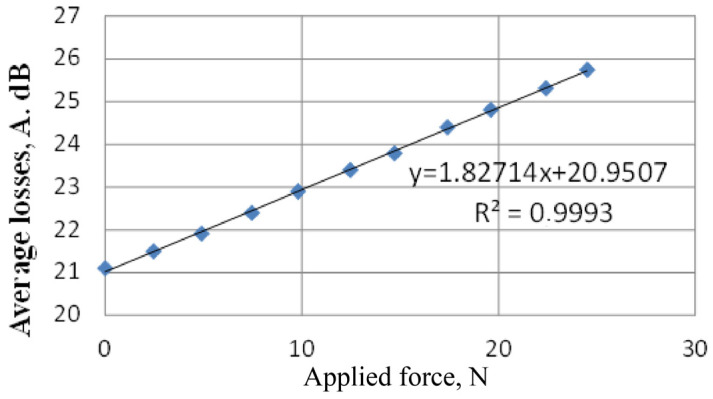
The relationship between the additional losses of the light wave and the applied force.

The experimental data were processed using the Wolfram Alpha (computational knowledge engine). The relationship between the additional losses of the light wave and the applied force was plotted, which is shown in Figure 6.

When the force was applied to the optical fiber, a microbend was formed, which led to the appearance of the photoelasticity effect. Accordingly, the higher the applied force was, the greater was the deformation of the OF and the higher was the additional loss of the transmitted light wave. The fiber-optic sensor had sufficiently high linearity and was capable of controlling with high accuracy the change in rock pressure on the elements of the mine support.

The following results were obtained when making automatic approximations.

1. 1.82714x+20.9507=y was the linear approximation.
2. $0.191111x^{3}-0.769524x^{2}+2.51127x+20.9205=y$ was the third-order (cubic) approximation.
3. $0.0904762P^{2}+1.55571x+21.0638=y$ was the second-order (quadratic) approximation.

Since the model with the lowest AIC (Akaike Information Criterion) was considered the best mathematical model, the losses in an optical fiber were better represented by a linear approximation, for which AIC was 7.25301, R^2^ = 0.980452 (determination coefficient).

Experiments were carried out to determine the additional power losses of optical radiation passing through a fiber-optic sensor at various applied forces. As a result, the laboratory sample of the fiber-optic sensor showed fairly high linearity. To achieve the accuracy of the results obtained, the number of necessary measurements was taken from the calculation of the Student’s coefficient 2.110 with a confidence interval of 0.93. The evaluation of the results obtained was carried out taking into account the smallest value of the information criterion Akaike. The measurement results were used to calculate the absolute error of 2.26 and the relative error of 8.833%.

**Figure 7:**
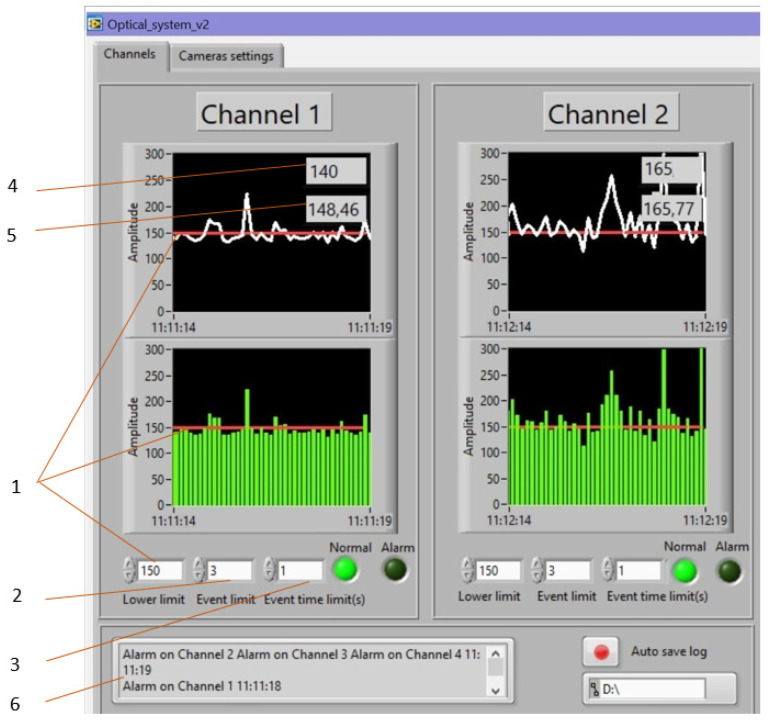
The appearance of a four-channel FAMS: (**1**) threshold value, (**2**) number of operations, (**3**) period of operation time, (**4**) average amplitude, (**5**) instantaneous amplitude, (**6**) response time fixation window.

One of the results of the study was the development of software designed to conduct pixel analysis of the change in the spot area and the intensity of the incident light wave on the surface of the TV matrix. The program analyzes the change in the pixel pattern and the color of the pixels from black to white. All changes were recorded in the computer memory and, based on the analysis, the result of the offset change was issued. Figure 7 shows screenshots, which show how the intensity of a light wave changes with an increase in additional losses in an optical fiber. The program can clearly track pressure fluctuations in real time and provide numerical values for its change. Figure 7 shows individual bursts, which are interference. The hardware-software complex can determine the received data and exclude false alarms of FAMS. Machine learning capabilities were used to control the shape of the light spot. This allowed FAMS to be trained, adapting it to any mining condition. The system can control the change in rock pressure and the displacement of rocks of the roof by changing the level of additional losses and changing the intensity of the light wave incident on the surface of the photodetector; the intelligent processing of the spot image allows one to track changes in the intensity of individual pixels.

There were four measuring channels in the interface of the hardware-software complex. Figure 7 shows the appearance of a four-channel FAMS.

FAMS has several settings, which are represented by buttons. There is a separate tab for the photomatrix settings. There are three windows for entering settings:

(1) Event limit shows the number of events within a specified time.
(2) Event time shows the response time or time period during which the measured parameter exceeds the upper limit.
(3) Upper limit shows the upper threshold of operation.

The hardware and software complex can simultaneously measure pressure parameters in four channels. It also has a built-in alarm function that is triggered when there is a sharp displacement of layers and a threat of collapse occurs. Each measuring channel is equipped with a separate collapse alarm. At the bottom of the program interface there is an alarm screen, where the zone of the expected collapse is indicated. Before starting work, the device was calibrated: The initial parameters were set. Then, the photomatrixes recorded the change in the shape of the light spot at the exit from the OF before and after applying pressure to the optical fiber. The additional loss of the light wave passing through the core of the OF increased proportionally with increasing pressure across the OF. The photomatrix recorded the change in the shape of the spot and transmitted the information to the computer, where the developed software made it possible to estimate the change in the area of the diffraction spot and compare it with the spot samples before mechanical action. All changes were recorded in the computer memory and, based on the analysis, the result of the pressure change by the OF was given.

The user interface program performed the following functions.

(1) It provided input (the first input of the hardware settings of the photomatrix through the device driver program, the second input of the numerical values of the settings of the hardware–software complex)/output (provided real-time, channel image output from channels, had indicators for outputting alarms and a schedule for logging channel activity) of data required to control the process, namely, to set the initial parameters.
(2) It performed processing of data entered by the user and received from photomatrixes from all connected zones in accordance with the control tasks.

**Figure 8:**
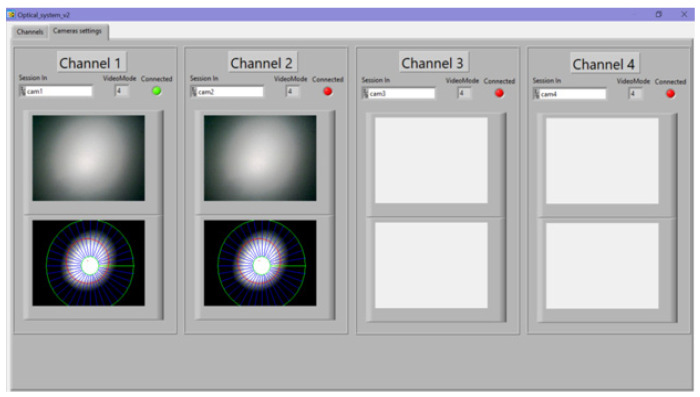
Change in the diffraction spot in the interface of the hardware–software complex.

Figure 8 shows the program window with images of light spots. The windows can simultaneously display four light spots in a calm and disturbed state under mechanical action on fiber-optic sensors. This function of the program was used for setting and calibrating it.

**Figure 9:**
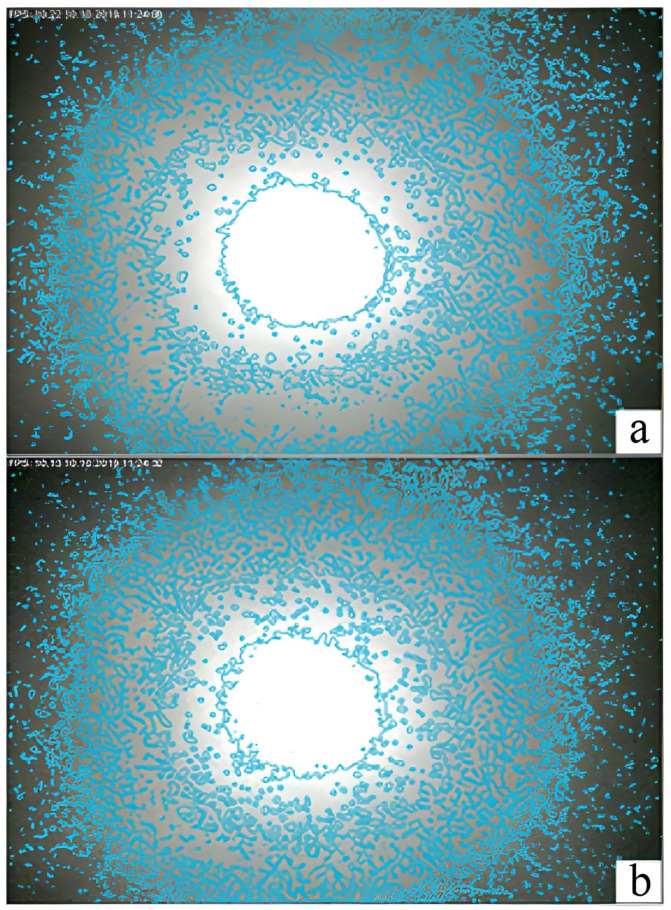
The shape of the light spots: (**a**) with a small mechanical impact and (**b**) with a greater mechanical impact.

The system can also change its sensitivity stepwise. Depending on the force acting on the OF, the light spot differs significantly. An example of a comparison of two light spots is shown in Figure 9. The resulting light spot is similar to the punch spot, with a brighter middle and darker outer regions. One can also see how the shape of the spot changes with the OF perturbation; the number of pixel changes and how they change colors can be seen in Figure 9.

During measurements, the diameter of the diffraction spot was compared and the pixels were calculated. If the number of black pixels decreased after changing the light mode, then the counter was triggered and the time was fixed, as well as sound being fixed. Thanks to this, this FAMS is still able to act as an alarm in the event of a sudden roof collapse.

**Figure 10:**
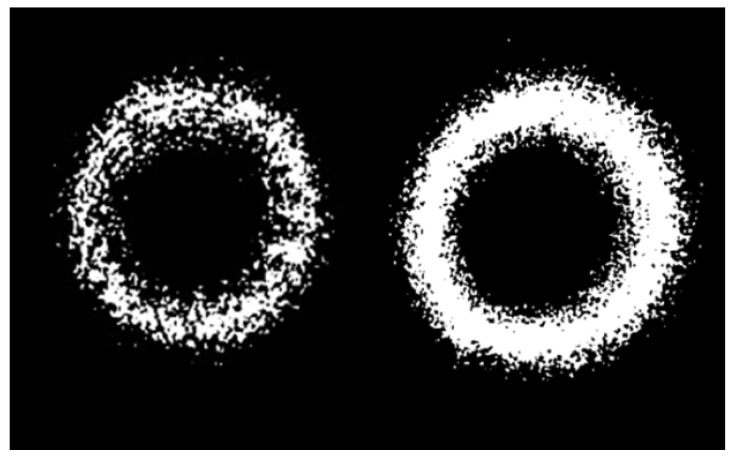
The dynamics of pixels’ growth under increased pressure on OF.

Figure 10 shows the situation with an increase in pressure on the OF. The white pixels separate from the main ring are the result of laser pulsation noise. The shape of the light spot differs from the original since the image has undergone software processing and the results of pixel analysis are presented on the screen.

**Figure 11:**
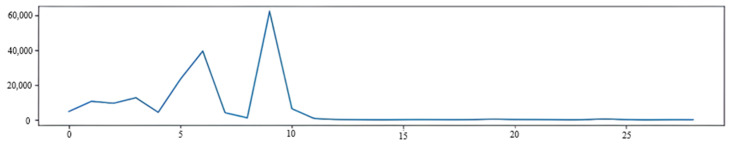
Plot of changes in the number of pixels in laboratory tests of FOS.

One of the features of the operation of the hardware–software complex is controlling the rate of changes in the derivative of the light wave intensity in time, as well as changes in the shape of the spot and the transition of pixels from white to black. The growth of white pixels can be caused by pulsation of the laser source, in which the wavelength of the light varies within 5 nm. The program recognizes noise since an increase in noise and an increase in pressure are different indicators of changes in the intensity of the light spot. The hardware–software complex allows one to numerically estimate the pressure level on the optical fiber when rocks are displaced. One can also watch the change in the light spot in real time. As the pressure increases, the number of white pixels increases. Figure 11 presents a graph on which the y-axis shows changes in the number of white pixels and the x-axis shows the number of frames. The graph illustrates the number of impacts on the OF per unit of time, leading to a change in the number of pixels. The graph was obtained in laboratory tests by short-term exposure on the OF. At the same time, the OF bent strongly several times, which was fixed by the program.

**Figure 12:**
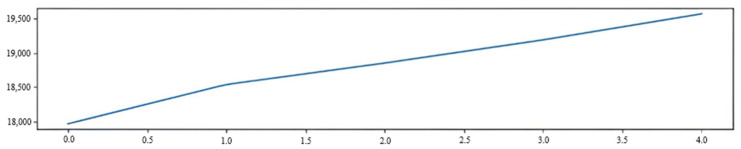
Plot of pixel increase during FOS lab tests.

The graph of a smooth increase in pressure on the OF during laboratory tests of FOS is shown in Figure 12. The screw of the clamp was twisted very slowly and smoothly. The load on the OF changed in the process of tightening the clamp screw from 0 to 30 N. The tightening force of the clamp screw was measured using a strain gauge. The clamping element had a cup shape, which made it possible to act on the OF simultaneously at two points, as shown in Figure 7. The clamping element formed a microbend with a radius of 1 mm. A microbend was formed in the OF, which led to the appearance of additional losses in the power of the light wave. The OF was located on a soft substrate, which prevented its crushing and destruction.

Figure 12 shows an increase in the number of white pixels. Accordingly, the higher the pressure on the OF, the more pixels change from black to white per unit of time.

**Figure 13:**
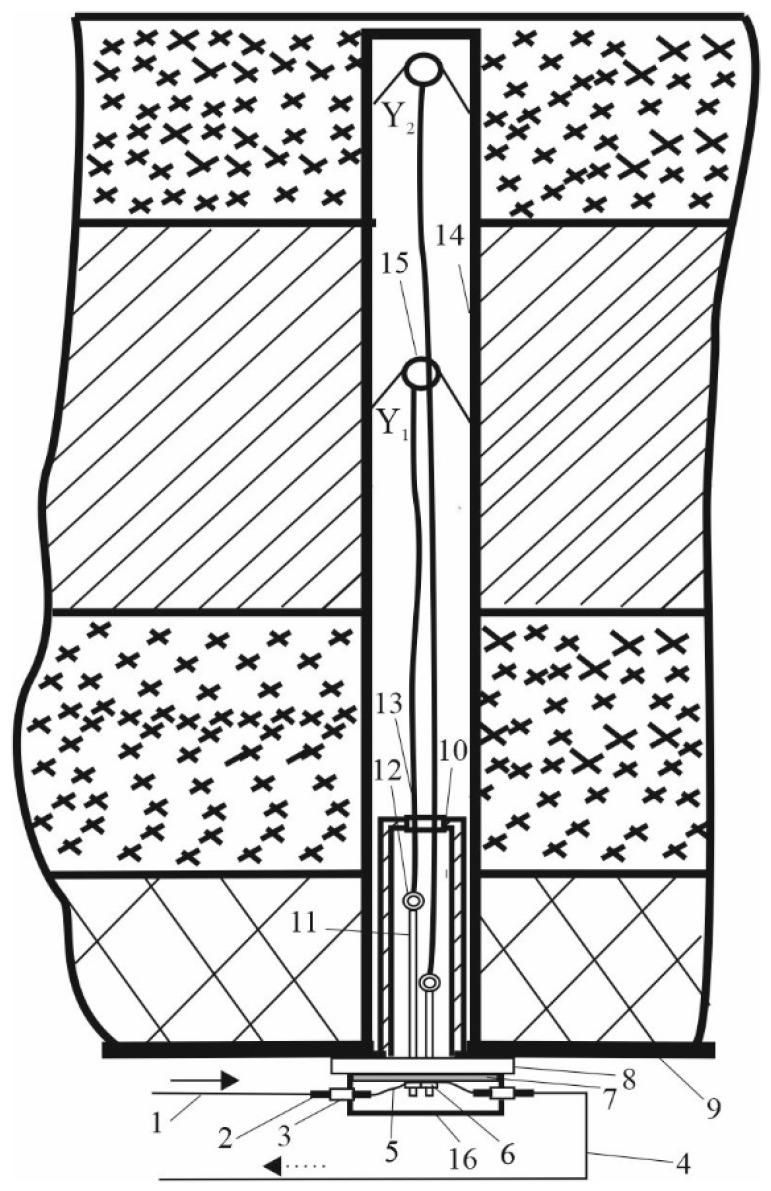
Installation of the two-level FOS: (**1**) fiber-optic patch cord, (**2**) duplex optical connector, (**3**) duplex optical adapter, (**4**) fiber-optic patch cord at the output, (**5**) sensitive element, (**6**) element of action on the optical fiber to create a microbend, (**7**) damper, (**8**) support metal disk, (**9**) mine ceiling, (**10**) guide sleeve for installation and fastening of the FOS in the borehole, (**11**,**12**) connecting pin with eyelet, (**13**) cable for attaching the clamp, (**14**) hole, (**15**) clamp, (**16**) base.

The result of the research was the developed scheme of a quasi-distributed FAMS. Pressure and rock displacement sensors were installed in the roof of the mine between the arched support frames. For this, a borehole with a diameter of 43–60 mm was drilled using a drilling tool to a depth of 6 m. The design of the mechanical part of the FOS and the process of its installation were similar to the well-known reference station, which is already used in the coal mines of ArcelorMittal Temirtau JSC. Therefore, the existing drilling equipment could be used and additional training of personnel was not required. The sensors had to be installed in the immediate vicinity of the long wall in two haulage drifts at a distance of 200 m from the long wall. This zone had a maximum displacement of layers and a change in the parameters of rock pressure, which caused deformation of the arched support. In total, one should install 20 sensors. One set of FAMS is capable of working with 60 sensors simultaneously in real time. The remaining sensors were placed in hazardous areas that were formed in the process of long wall mining and its motion through the coal seam. A two-layer FOS was placed inside the borehole and wedged, as shown in Figure 13.

The main elements of the FOS were the duplex fiber-optic patch cord (1) connected to the sensor input, the duplex optical connector (2), the duplex optical adapter (3), the duplex fiber-optic patch cord (4) connected to the sensor output, the sensitive element (5), an element of action on the OF to create a microbend (6), the damper (7), the support metal disk (8), the mine ceiling (9), the guide sleeve for installation and fastening of the FOS in the borehole (10), the connecting pin with an eyelet (11), the cable for attaching the clamp (13), the hole (14), the clamp (15), and the housing cover for the FOS (16). The sensing element (5) was made of OF and was placed between the element of action on the OF to create the microbend (6), damper (7), and support metal disk (8). When the layers were displaced, the walls of the borehole (14) were destroyed and the clamp (15) began to shift in the direction of the motion of the layers of the rock mass. When the layers were displaced, the rock pressure on the arched support elements changed. Since the clamp (15) was mechanically connected to the eye of pin (12) by means of the cable (13), which was pulled when the clamp moved, this caused a mechanical effect on the sensing element (5) through the element of action on the OF to create the microbend (6). The sensing element was located on a soft rubber damper lying on the surface of the supporting metal disk (8). The more the clamp (15) was displaced, the more pressure was exerted on the sensing element (5), which caused a photoelastic effect and changed the properties of light passing through the OF core.

The first level was designated Y_1_ and was located at a depth of 2 to 3 m. The second level was designated Y_2_ and was located at a depth of 4 to 5 m. The holes were drilled with a step of 20 to 50 m, taking into account the geotechnical state of the upper working layers and their tendency to displacement.

One FOS was connected using a duplex fiber-optic patch cord, which contained two OFs. Accordingly, it was necessary to allocate two OFs for one input. One OF was used to supply the light wave to the FOS, and the second transferred the light wave from the sensor to the data processing unit. The light wave passed through the FOS and returned to the photomatrix, carrying information about changes in the measured parameters. The maximum distance from the data processing unit to the FOS was 20 km. The light wave passed through the sensing element (5) and returned to the data processing unit, carrying the necessary information. A television matrix was installed in the data processing unit. The FOS used a semiconductor laser with a wavelength of 650 nm and a power of 30 mW. The FOS was designed for ease of installation and to be maintenance-free. The sensor was energetically passive and did not pose a threat to the explosion of the mine atmosphere since it did not use electric current in its operation. The cost of the proposed two-level FOS was about USD 90. In terms of mass production, it will be significantly lower. Accordingly, the difference in cost between a mechanical testbench and the FOS is approximately USD 30, which is quite acceptable. The financial costs for eliminating the consequences of the destruction of the arch support and clearing debris are much higher than the costs of installing the FAMS. The FOS was connected using standard SC or FC connectors and adapters with a 2.5-mm UPP polished ferrule. The use of connectors instead of welded joints was a necessary measure since welding in coal mines is dangerous because of the possible sudden release of coal dust and methane gas and is, therefore, prohibited. The proposed solution guarantees complete explosion safety during installation work.

**Figure 14:**
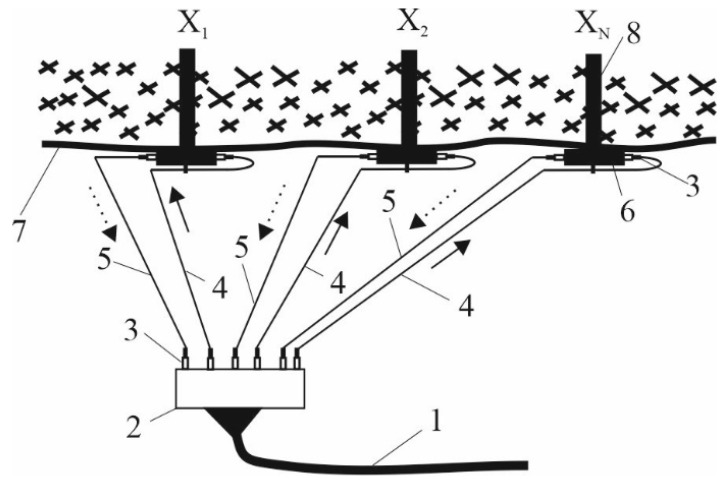
FOS connection diagram of quasi-distributed FAMS: (**1**) fiber-optic cable, (**2**) optical cross, (**3**) optical connectors, (**4**,**5**) fiber-optic patch cords, (**6**) fiber-optic sensor, (**7**) mine working ceiling, (**8**) borehole.

Figure 14 shows the FOS wiring diagram for a quasi-distributed FAMS. The system consists of an armored fiber-optic cable (1), which is used to connect the sensors to the data processing unit. For each of the two-level sensors, one module is used, consisting of four OFs of the G.652 standard (Corning, USA) with a diameter of 9/125 μm. The cable is connected to the optical distribution frame (2). The sensors are connected using detachable connections, in the role of which optical connectors are involved (3). Fiber-optic patch cords (4) extend from the cross section (2), through which the light wave is fed to the FOS, and then, after passing through the sensitive element, returns to the fiber-optic patch cords (4). The direction of the light motion through the FOS is shown by arrows. The FOS (6) is installed in a hole (8) drilled in the ceiling of a mine (7), connected to an optical distribution frame (2) using optical connectors (3) and patch cords (4 and 5).

To connect a group of up to 30 two-level FOSs, one armored fiber-optic cable with 120 cores can be used. Since, for safe operation near the bottomhole space, it is necessary to install 20 sensors in one haul road, two cables must be used. Each can be connected to up to 30 sensors. In practice, 20 sensors are sufficient per one haul road. The remaining 20 sensors can be installed in potentially hazardous locations.

To connect the sensors and the FAMS data processing unit, an armored fiber-optic cable was used, which was laid along the side of the mine with the cables of the electric network and mine telephone connection. Each roadway will have its own individual cable for connecting the optical distribution frame. As practice has shown, reference stations are installed after 50 m (or, more rarely, after 30 m). This is quite enough to control the rock pressure.

## 4. Discussion

At the moment, only laboratory experiments have been carried out; accordingly, after full-fledged production tests, appropriate adjustments will be made that will improve the design of the FAMS. To carry out full-fledged production tests, it is necessary to obtain several permits from the ArcelorMittal Coal Department, which takes significant time and organizational effort. Initially, the hardware-software complex was created for four channels. In the real conditions of the mine, more than 20 channels are required to effectively control the rock pressure of the working, depending on its geotechnical parameters. The disadvantages of the proposed sample of the FAMS are the occurrences of interference associated with the instability of the laser, which in some cases caused a false warning about a sharp displacement of rocks and the formation of a dangerous collapse of a mine working. This problem was solved by reducing the sensitivity of the FAMS. Accordingly, in subsequent work, a laser with smaller deviations, of 650 nm ± 5 nm, will be used and experiments will be carried out using a wavelength of 850 nm. This will reduce the level of interference, as well as increase the distance from the FOS to the data processing unit. Therefore, the modernization of the hardware–software complex is required to expand the capabilities.

An important point is the stability of the laser. The laser pulsation greatly affects the changes in pixels, and the higher pulsation causes more interference. Therefore, during the experiment, lasers with a wavelength deviation exceeding 10% were rejected since they introduced significant interference that disrupted the operation of the hardware–software complex and caused false values of the measured values. The stability of the laser to a greater extent affects the stability of the hardware–software complex as a whole. The environment temperature where the experiments were carried out was regulated at the level of 20–26 °C to detect temperature interferences, which were not recorded. The temperature was controlled by an air conditioner operating in heating mode. The temperature was changed intentionally to fix temperature disturbances, which may appear when the temperature rises above 30 °C. According to the safety requirements, an artificial microclimate is maintained in the mine working and the temperature is regulated at the level from 18 to 26 °C. Therefore, the specified temperature range was quite acceptable. To improve the efficiency of the hardware and software complex and reduce the impact of various kinds of interference occurring in the measuring channels, it is necessary to further use the capabilities of neural networks. This will allow for a more accurate assessment of the data obtained.

## 5. Conclusions

Fiber-optic sensors for monitoring displacement will allow monitoring the pressure change of rocks in the working when changing pressure on the elements of the mine support, which will reduce the threat of sudden rock collapse. It is also possible to achieve full automation of the process of controlling rock displacements in the development, to reduce significantly the proportion of manual labor, and to eliminate errors in measurements. The work aimed at developing digital technologies and their implementation in mining. Timely warning of an impending collapse threat will increase the level of safe mining operations. With timely notification of increasing rock pressure and displacement of the roof rocks, it will be possible to take appropriate measures to strengthen the support and to prevent its destruction. This will reduce significantly the cost of supporting mine workings and ensure safe operation. The FOS has a sufficiently high linearity of characteristics and fully meets the requirements of explosion safety. Unlike the well-known systems based on an optical interferometer, fiber Bragg gratings, and long-period fiber gratings, the presented design differs in a lower cost of one measurement point since it does not use expensive equipment (optical time domain reflectometer or spectrum analyzer), which makes it more suitable for introducing in coal mines that are hazardous for explosion of methane gas. In contrast to the existing FOS designs based on an optical interferometer, the proposed FAMS is less dependent in terms of interference with temperature changes since there is no interference spot. It is known that to change the beam path of an optical interferometer a change within 1 °C is sufficient since it is quite sensitive to temperature fluctuations. If we take into account that the length of the fiber-optic measuring channel can reach 10 km, then the temperature interference in the interferometer will become significant. The main advantage of the proposed FAMS is its explosion safety since electrical signals are not used in the measuring channels. This allows us to count on its implementation in the production process in accordance with the requirements of industrial safety. Another important point is the simplicity of design and the low cost of one measuring point. The advantages of this system are that the FOS is located in an explosive environment and the data processing unit is located in the near-shaft yard or on the surface. This is ensured due to the properties of the optical fiber, which is capable of transmitting light signals over a considerable distance with minimal losses compared to IMS using electrical signals. When the OF has a break, the system automatically gives a signal about the loss of communication with the FOS, while the rest of the sensors remain in operation. The proposed FAMS is built on a quasi-distributed scheme, capable of operating in two main modes of measurement and signaling. In the event of a sharp change in rock pressure and displacements of rocks of the roof of the mine, the FAMS warns of the danger by giving a signal to the operators.

## Data Availability

Not applicable.
